# Reverse Adverse Immune Microenvironments by Biomaterials Enhance the Repair of Spinal Cord Injury

**DOI:** 10.3389/fbioe.2022.812340

**Published:** 2022-05-13

**Authors:** Hengyi Wang, Yuanliang Xia, Baoqin Li, Yuehong Li, Changfeng Fu

**Affiliations:** Department of Spine Surgery, The First Hospital of Jilin University, Changchun, China

**Keywords:** spinal cord injury, immune microenvironment, immune cell, cytokines, reactive oxygen species, extracellular matrix

## Abstract

Spinal cord injury (SCI) is a severe and traumatic disorder that ultimately results in the loss of motor, sensory, and autonomic nervous function. After SCI, local immune inflammatory response persists and does not weaken or disappear. The interference of local adverse immune factors after SCI brings great challenges to the repair of SCI. Among them, microglia, macrophages, neutrophils, lymphocytes, astrocytes, and the release of various cytokines, as well as the destruction of the extracellular matrix are mainly involved in the imbalance of the immune microenvironment. Studies have shown that immune remodeling after SCI significantly affects the survival and differentiation of stem cells after transplantation and the prognosis of SCI. Recently, immunological reconstruction strategies based on biomaterials have been widely explored and achieved good results. In this review, we discuss the important factors leading to immune dysfunction after SCI, such as immune cells, cytokines, and the destruction of the extracellular matrix. Additionally, the immunomodulatory strategies based on biomaterials are summarized, and the clinical application prospects of these immune reconstructs are evaluated.

## 1 Introduction

### 1.1 Review of Spinal Cord Injury

Spinal cord injury (SCI) is the main type of central nervous system (CNS) injury and can lead to paraplegia or quadriplegia and affect the quality of life of patients (Li et al., 2019a). According to statistics, the total global incidence of SCI is 10.5 per 100,000 people, resulting in an estimated 768,473 new cases of SCI annually worldwide (Kumar et al., 2018). There are two types of SCI injury: primary and secondary. Primary damage can cause immediate neuronal death and tissue damage, and secondary damage usually leads to permanent dysfunction. Necrosis and apoptosis of nerve cells occur after spinal cord injury (Hachem et al., 2017). The inhibition of nerve regeneration in the disease is due to the inflammatory response in the microenvironment, the formation of glial scars, the loss of nutritional factors and myelin proteins, the interruption of blood flow, the loss of spinal cord tissue, the formation of cavities, and other adverse factors (Hellenbrand et al., 2019; Li et al., 2020a; Perrouin-Verbe et al., 2021). Therefore, neuromorphic repair and functional recovery after spinal cord injury remain challenging for patients and clinicians.

### 1.2 Spinal Immune Defense

The CNS has its own immune defense system, among which the main immune defense cells are the resident microglia (Streit JRC et al., 2016), whose activation is closely related to the cascades of damage and recovery after CNS trauma. Activated microglia recruit peripheral monocytes to the damaged area (Devanney et al., 2020). The differential polarization of microglia affect the development direction of the local inflammatory response after SCI. After SCI, most microglia are polarized to the M1 phenotype, where pro-inflammatory mediators such as tumor necrosis factor-α (TNF-α), inducible nitric oxide synthase (iNOS), cyclooxygenase-2 (COX-2), and interleukin 1β (IL-1β), are released in large quantities. These factors prolong and aggravate the neuro-inflammatory process, leading to the death or dysfunction of neighboring neurons, which is not conducive to the repair of SCI (Xiao et al., 2021). In contrast, promoting the polarization of M2 microglia can reduce the inflammatory response at the injury site and promote the repair of SCI (Zhang et al., 2019a; Xiang Zhou et al., 2020). Because of the characteristics of different polarization directions, regulating the polarization of microglia toward inhibiting the inflammatory phenotype has become a new target for treating SCI. This approach will be reviewed in detail in the following chapters.

Astrocytes are the resident cells of the CNS and can respond to danger signals, secrete chemokines and cytokines, and recruit immune cells along with other chemokines released by injury-induced inflammation, such as monocytes, neutrophils, and lymphocytes (Ji et al., 2021). Astrocytes can be activated into neurotoxic type A1 and neuroprotective type A2. The different activation directions hold great significance for the progression of SCI, with both having advantages and disadvantages. Therefore, the targeted activation of astrocytes to form a favorable microenvironment to combat secondary injury is crucial for the recovery of SCI (Li et al., 2020b). Some experiments have shown that treatments such as drugs and mesenchymal stem cell (MSC) exosomes can effectively inhibit the activation of type A1 astrocytes, reduce the size of the lesion, inhibit the formation of glial scars, reduce inflammation, and promote axis regeneration (Wang et al., 2018; Zou et al., 2021). Normally, the spinal cord is immune protected because of the presence of the blood spinal cord barrier (BSCB). The BSCB is a physical and biochemical barrier between the systemic circulation and the CNS, which serves to maintain the homeostasis of the spinal cord microenvironment (Blume et al., 2020). However, damage caused by various factors can destroy this barrier, allowing immune cells and other substances to enter the injury site (Ahmed et al., 2018a). The increased permeability of the BSCB leads to the formation of spinal cord parenchymal edema, and allows peripheral circulating inflammatory cells to enter the spinal cord parenchyma, leading to further SCI. During this process, the unaffected spinal cord will also be damaged and the destruction of the BSCB is related to multiple sclerosis (MS), amyotrophic lateral sclerosis (ALS), SCI, and many other acute and neurodegenerative diseases (Bartanusz et al., 2011). As the BSCB plays a vital role in the homeostasis of the spinal cord, its use as a therapeutic target will become a hot spot for treating SCI in the future. Some studies have shown that the inhibition of matrix metalloproteinase (MMP) by drugs and cell transplantation to prevent a decrease in BSCB permeability can slow down the secondary damage of SCI and improve the prognosis (Garbuzova-Davis et al., 2017; Zhang et al., 2017; Zheng et al., 2020).

### 1.3 Treatment Strategies and Challenges

Spinal cord mechanical injury leads to rupture of nerve axons, causing severe nerve damage, with no effective clinical treatment proposed as yet (Arac et al., 2021). Early anatomical changes are the main characteristics of primary injury, while secondary injury is an important factor affecting the degree and effect of SCI recovery. The destruction of the BSCB causes the inflammatory cells and related factors in the peripheral circulation to enter the spinal cord, which further aggravates the inflammatory response and the death of nerve cells at the injury site (Lee et al., 2012). Secondary injury as a cascade is a process consisting of spinal cord hemorrhage, ischemia, the production of reactive oxygen species (ROS), lipid peroxidation, ion imbalance, and cell excitotoxicity, which can occur within a few minutes after trauma (Ahuja and Fehlings, 2016). The treatment of SCI is limited, and neither medication nor surgical treatment has achieved satisfactory results. Although drug treatments such as hormones and cytokines show positive effects, it is difficult to maintain drug concentration and dosage. Although surgery can help maintain stability and reduce secondary damage, it cannot promote nerve regeneration, bridge the space for damaged nerves, or fully restore nerve function (Bedir et al., 2020). Recently, new therapies, such as cell or nerve transplantation, have become research hotspots (Gao et al., 2020). The lost glial cells and neurons can be replaced by transplanted cells to produce a growth-permissible extracellular matrix to affect cell differentiation and survival, and secrete key neurotrophic factors that regulate the local microenvironment (Zavvarian et al., 2020). Cell therapy includes the transplantation of olfactory ensheathing cells (Pourkhodadad et al., 2019), Schwann cells (SCs) (Mousavi et al., 2019), adipose-derived stem cells (ADSCs) (Yuan et al., 2021), neural stem cells (Ceto et al., 2020), induced pluripotent stem cells (IPSC) (Kajikawa et al., 2020), and MSCs (Liau et al., 2020), with the aim to create a good environment for nerve regeneration. However, cell therapy is not without limitations. For example, the low survival rate of transplanted cells, the shortage of donors, transplant rejection, and ethical issues have restricted the application of autologous or allogeneic nerve transplantation for treating SCI (Gazdic et al., 2018; Cofano et al., 2019; Csobonyeiova et al., 2019).

**SCHEME 1:**
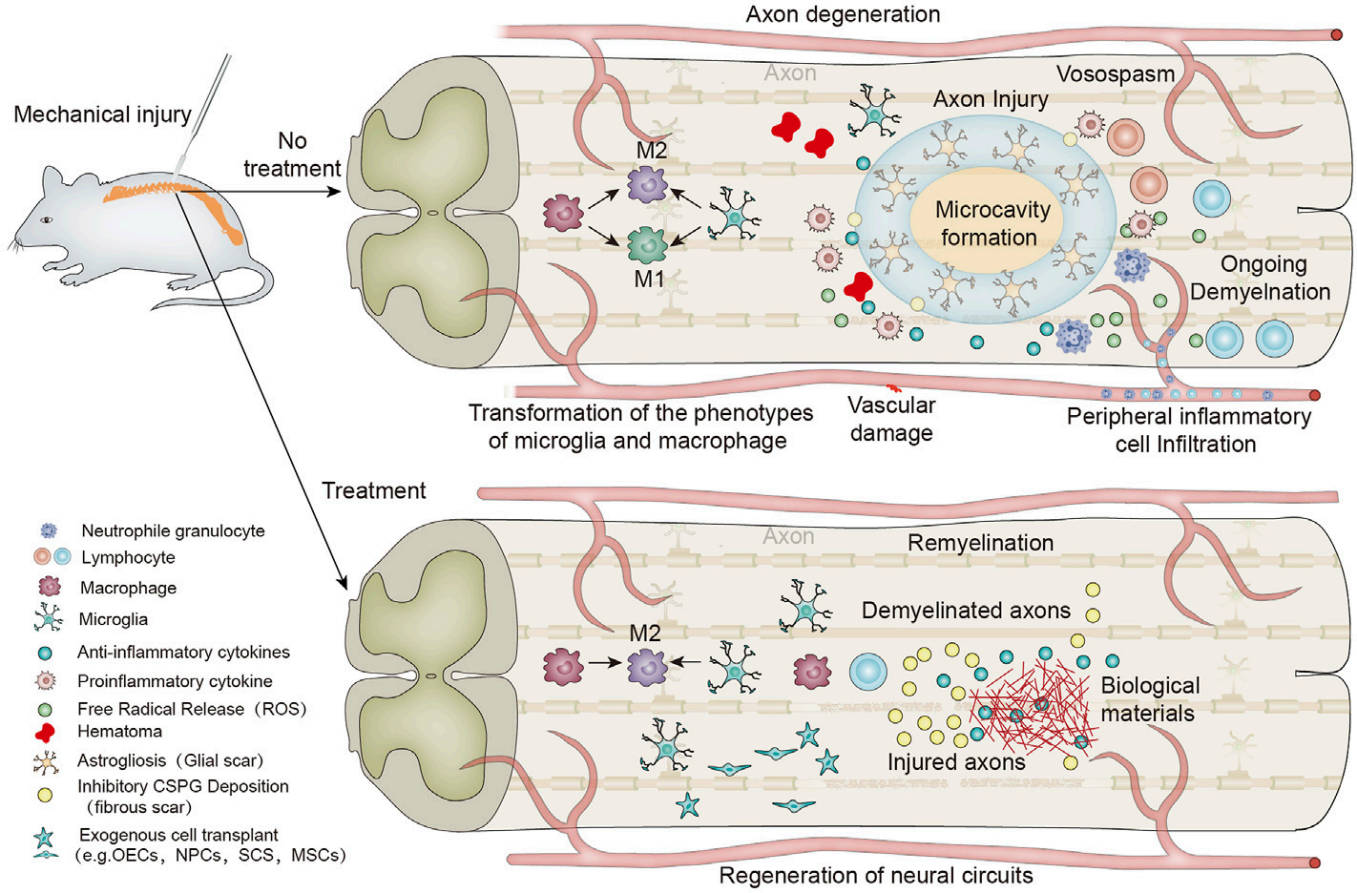
Schematic diagram of biomaterials regulating the local immune microenvironment after spinal cord injury and promoting nerve regeneration and myelination.

In recent decades, the application of neural tissue engineering technology based on biomaterials for treating SCI has been extensively studied (Abbas et al., 2020; Bedir et al., 2020; Xi et al., 2020). Seed cells, soluble biologically active molecules and biological materials are important components of neural tissue engineering. Commonly used seed cells for neural tissue engineering include SCs, stem cells and olfactory ensheathing cells; soluble bioactive molecules promote cell proliferation and differentiation; and biomaterials are used as carriers for seed cells and soluble bioactive molecules to construct and connect nerve tissue vacant due to injury. Progress has been made not only in the fields of axon regeneration but also in reduction of scar formation, inhibition of inflammation, and cell implantation (Courtine and Sofroniew, 2019). The goal of biomaterial-based neural tissue engineering is to produce standard functional structures to support and guide the regeneration of nerve tissue in damaged spinal cord defects or gaps. The ideal nerve tissue scaffold should have good biocompatibility, proper biodegradability, excellent mechanical strength, porosity, and electrical conductivity. Its porous properties can support the connection with the extracellular matrix, allowing the exchange of nutrients and waste (Zimmermann et al., 2021). So far, different types of polymer materials have been used to develop neural tissue engineering scaffolds, including various natural polymers, such as hyaluronic acid (HA) (Ding et al., 2019), chitosan (Cs) (Sun et al., 2019a), and collagen (Chen et al., 2017), and various synthetic polymers, such as polycaprolactone (PCL) (Zhou et al., 2018a), poly (lactide-co-glycolide) (PLG) (Anzalone et al., 2018), poly-L-lactic acid (PLLA) (Fitzgerald et al., 2018), and poly (ethylene glycol) (PEG) (Lu et al., 2018). At present, most of the treatment methods of SCI based on biological materials are still in the experimental stage and have not yet become conventional clinical treatment methods. Despite this, it is gratifying that many studies have shown significant progress in biomaterials, stem cell transplantation, immune regulation, and nanotechnology in spinal cord regeneration therapy (Ashammakhi et al., 2019). The difficulty of SCI treatment is greatly increased because of the complicated pathophysiological changes of SCI. To obtain better treatment effects, promote nerve regeneration after SCI, and improve the prognosis of SCI, various combination treatment strategies have emerged consecutively, including biomaterials and soluble biomolecules (Chen et al., 2018), biomaterials and cells (Mosley et al., 2017), biomaterials, soluble biomolecules and cells (Führmann et al., 2018), and other combined treatment methods. The emergence of these therapeutic methods has laid a good foundation for treating SCI. This paper summarizes the previous research results and development advantages of biomaterials to reverse the adverse immune microenvironment and promote SCI repair (Scheme 1 and Table 1).

**TABLE 1:** Physical properties and functions of polymer biomaterials.

Materials	Carrying substance	Fabrication technology	Properties	Function	References
Chitosan-FPHS	**—**	Take off the acetylation	Cytocompatibility Biodegradability non-toxic	Promote macrophage polarization to M2 phenotype, Reduce scar formation, Inhibit astrocyte proliferation	Chedly et al. (2017)
NHC	**—**	The physical blending	Excellent mechanical strength and porosity	Increase the ratio of M2 macrophages and promote angiogenesis, axon growth and neurogenesis	Li et al. (2020a)
IOA	**—**	Enzymatic hydrolysis	biocompatibility	Promote macrophage polarization to M2 phenotype	Cornelison et al. (2018)
OPF+	Schwann cells	Azeotropic distillation, crosslinking	Excellent compression and bending modulus	Promote macrophage polarization to M2 phenotype, Reduces stromal scarring, cyst formation, astrocyte reactivity, CSPG deposition, and myelin sheath debris	Hakim et al. (2015)
PLG	IL-10,NT-3, IL-4	Gas foaming/particulate leaching, Electrospinning technology	biodegradable	Alter macrophage phenotype, Promote axon growth and function recovery, Inhibits the release of inflammatory factors	(Park et al. (2018),Smith et al. (2020))
Poly(lactide-coglycolide) NPs	**—**	Gas foaming/particulate leaching, Steam method	biodegradable	The number of M2 macrophages was up-regulated, Reduce gelatinous scars, Axon regeneration, Recovery of motor function	Park et al. (2019)
carbomer/agarose/polyethylene glycol (PEG) based hydrogel	HMSC	**—**	Biocompatible, biodegradable	Promote macrophage polarization to M2 phenotype, Protective neuron	Papa et al. (2018)
CS	MSC	Chemical extraction, freeze drying	Nerve guide	Promote macrophage polarization to M2 phenotype, Protective neuron, Reduce gelatinous scars, Inhibit astrocyte proliferation	Peng et al. (2018)
PLLA	Ibuprofen and T3	Electrostatic spinning	Biocompatibility biodegradability	Reduce the number of M1-type macrophages	Bighinati et al. (2020)
MoS2@PEG	ET	Microwave-assisted hydrothermal method	Biocompatibility biodegradability	Promote macrophage polarization to M2 phenotype, Protective neuron, Improved motor function	Sun et al. (2019b)
DS	MH	self-assembly	Biocompatibility	Promote macrophage polarization to M2 phenotype, Improved motor function	Wang et al. (2017a)
Type I collagen	**—**	Freeze drying	Biocompatibility, low antigenicity biodegradability	Reduce phagocyte recruitment	Snider et al. (2017)
Type I collagen	NT-3	Freeze drying	Biocompatibility, low antigenicity biodegradability	Reduced macrophage/microglia activity, Promote axon growth, Reduce scar formation	Breen et al. (2016)
PTNP	Piceatannol spleen tyrosine kinase inhibitor superparamagnetic iron oxide	Ultrasonic fusion Freeze-thaw cycle	Biocompatibility	Reduce neutrophil infiltration	Tang et al. (2019)
DOX-hyd-BSA NPs	DOX	Desolvation	Biocompatibility	Reduce neutrophil infiltration	Zhang et al. (2019b)
ASC	BMSC	**—**	Biocompatibility	Reduce T lymphocyte recruitment, Promote functional recovery	Wang et al. (2017b)
CCH	Serp-1	Remove the ion Freeze drying	Biocompatibility, low antigenicity biodegradability	Reduce T lymphocyte recruitment	Kwiecien et al. (2020)
PCL/BSA	DEXP	Electrospinning fibers	Good mechanical properties, Biocompatibility Biodegradability The hydrophilic	Inhibit astrocyte proliferation, Reduce oligodendrocyte apoptosis	Rasti Boroojenia et al. (2018)
MCM	IL-10	**—**	Biological adsorption, Good solubility	Inhibit inflammatory cytokines, Promote anti-inflammatory cell proliferation, Allows axons to grow	Hellenbrand et al. (2019)
MSaP-aL/p	Il-4 plasmid liposomes	Electrospinning technology	Acid responsiveness	Promote the secretion of anti-inflammatory factors, Promote macrophage/microglia M2 polarization, Inhibit astrocyte activation	Xi et al. (2020)
PCL/fibrin gel	MPSS	Nano precipitation technology	Biodegradability Locally sustained drug release	Inhibit IL-6 and IL-1β release	Karabey-Akyurek et al. (2017)
IKVAV PA	BDNF	Automatic solid phase synthesis method	Biocompatibility Biodegradability	Inhibit inflammation and astrocyte proliferation	Hassannejad et al. (2019)
SF/AGs/GDNF	GDNF	**—**	Low immunogenicity	Inhibit il-1 β, IL-6 and TNF-α release	Jiao et al. (2017)
PLGA	**—**	**—**	Biodegradability	Reduce CSPG accumulation	Jeong et al. (2017)
Ac-DEX	PTX	microprecipitation method	Good release rate	Reduce CSPG accumulation	Liu et al. (2018)
AST-PCL	AST	**—**	**—**	Down-regulation of MMP-9 secretion, reduction of neutrophil infiltration	Zhang et al. (2019c)
PEG	**—**	Water/oil emulsion method, free radical polymerization	The hydrophilic	Inhibits ROS production	Luo et al. (2002)
PSA	MH	Acylation reaction, self-assembly	Biocompatibility	Antioxidation, Anti-inflammatory effects	Wang et al. (2019)
PCL	MLT	Electrospinning technology, Multilayer molding process	Excellent mechanical properties, porosity	Correcting mitochondrial function reduces oxidative stress	Qian et al. (2018)
MnO^2^ NPs	MnO^2^	Crosslinking, biomineralization	Biocompatibility	Inhibits ROS production	Li et al. (2019c)
SeNPs@GM1/TMP	Selenium, GM1, TMP	**—**	**—**	Inhibits ROS production	Rao et al. (2019)

IL-4, Interleukin-4; IL-10, Interleukin-10; NT-3, Neurotrophin-3; hMSC, humanbonemarrow-deftvedmesenchymalstemcell; MSC, mesenchymal stem cell; BMSC, bone marrow stromal cells; T3, triiodothyronine; ET, etanercept; DS, dextran sulfate; MH, minocycline hydrochloride; DOX, doxorubicin; Serp-1, serine protease Inhibitors-1; DEXP, dexamethasone sodium phosphate; MPSS, methylprednisolone sodium succinate; BDNF, brain derived neurotrophic factor; GDNF, Glial cell line-Derived Neurotrophic Factor; PTX, paclitaxel; AST, astragaloside; MLT, melatonin; TMP, tetramethylpyrazine; GM1, gangliosides.

## 2 Biological Materials for Regulation of Immune Cells in the SCI Microenvironment

### 2.1 Biomaterials That Regulate the Polarization of Macrophages/Microglia

Macrophages are derived from the mononuclear macrophage system *in vivo* and are an important component of the immune system, playing an indispensable role in the body. Their plasticity enables macrophages to exhibit various polarization states *in vivo*, predominantly proinflammatory and anti-inflammatory. In contrast, microglia originate from the embryonic yolk sac and enter the brain early in embryonic development. As a resident immune cells in the CNS, they play a vital role in neuronal development, maintenance of nervous system homeostasis, and immune defense (Xu et al., 2021a). However, their phenotypic change is a double-edged sword. The imbalance in the number and proportion of microglia with different phenotypes is disastrous for the prognosis of SCI. Microglia and infiltrating macrophages play a major regulatory role in the local immune and inflammatory responses after SCI (Kroner and Rosas Almanza, 2019). Relevant studies have shown that microglia and macrophages are rapidly induced and recruited to the injury site after injury in the acute phase of SCI, but are dominated by pro-inflammatory M1 phenotypes, with few anti-inflammatory M2 phenotypes (Qichao et al., 2020). However, excessive inflammatory factor intervention is not conducive to functional recovery in advanced SCI. Therefore, regulating the polarization of microglia/macrophages can be used as an effective treatment for SCI.

#### 2.1.1 Biomaterials Directly Regulate Polarization

Cs is a natural polysaccharide polymer with biocompatibility, biodegradability, renewability, and nontoxicity, as well as unique properties such as anti-inflammatory, antibacterial, and antimicrobial effects (Yao et al., 2018). Therefore, Cs is widely used in drug-carrying engineering, tissue engineering and other applications. Chedly and other studies have shown that Cs-fphs as a substrate can directly affect the polarization of macrophages, and after implantation, can regulate the inflammatory response at the damaged site by promoting the polarization of macrophages to the anti-inflammatory M2 phenotype (Chedly et al., 2017).

**FIGURE 1:**
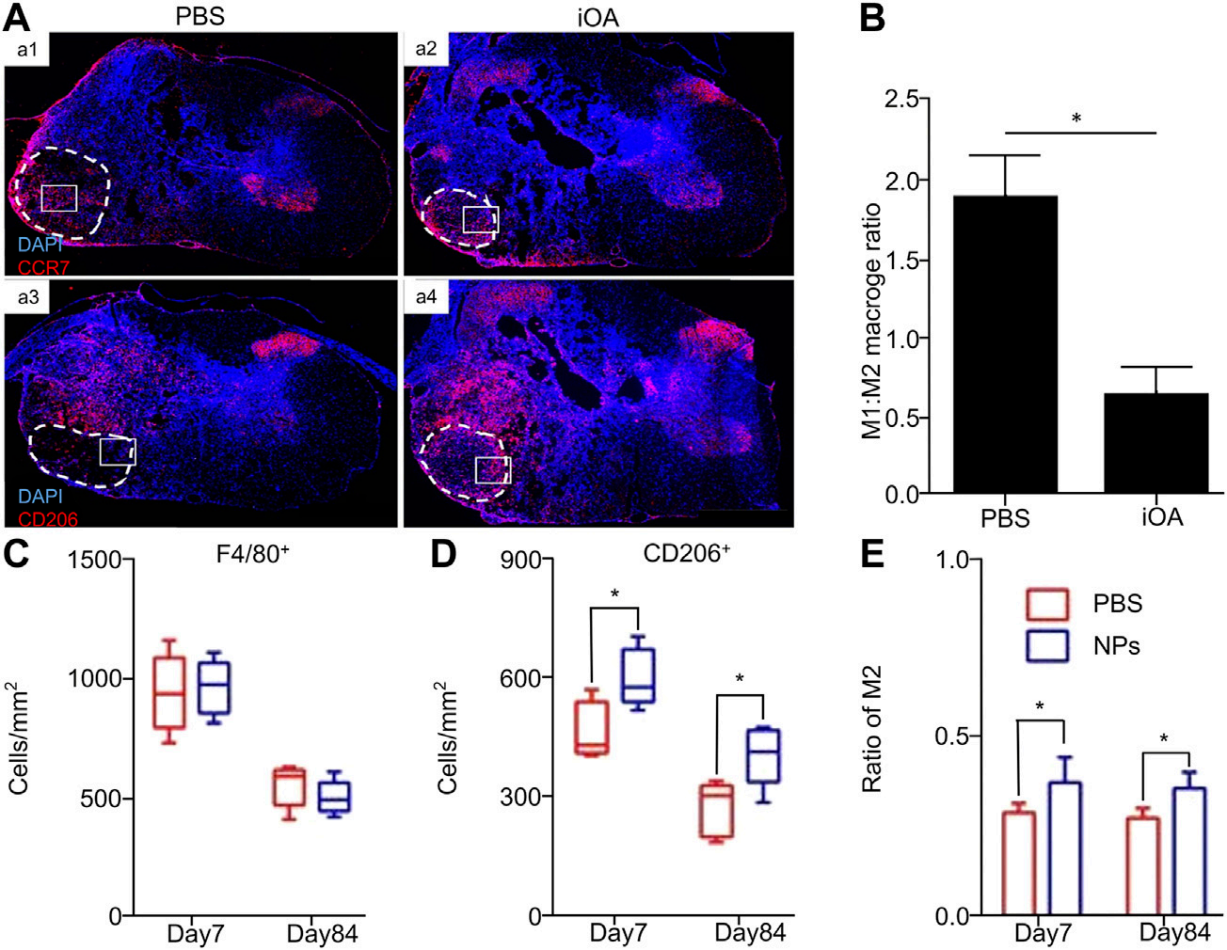
Biomaterials directly regulate the polarization of macrophages/microglia. **(A)** The animals were treated with PBS (a1 and a3) and iOA hydrogel (a2 and a4) for 1 week, and the cross-section of the spinal cord was stained for macrophage markers. CCR7 is stained for M1 phenotype cells, and CD206 is stained for M2 phenotype cells. **(B)** The ratio of M1 to M2 phenotypic cells changed from 1.88 ± 0.406 for PBS control to 0.635 ± 0.085 for iOA, a three-fold decrease (*p < 0.05; n = 4). **(C)** The density of total F4/80 + macrophages in the lesion. **(D)** Quantitative analysis showed that NPs up-regulated the expression of CD206 + cells. **(E)** The ratio of M2 to the total number of macrophages was significantly up-regulated in the NP group. Reproduced with permission from (60, 63).

Hydrogel is an effective method used to fill the voids of SCI. It has good interstitial compatibility and can be used as a scaffold to support axon growth (Papa et al., 2021; Xu et al., 2021b). Additionally, its natural properties can mimic the biological properties of the extracellular matrix (ECM) (Li et al., 2012). Li et al. designed a nanofiber-hydrogel composite material combining a fiber surface and hydrogel network interface. The material included PCL nanofiber-Ha hydrogel (NHC) and showed good biological phase, capacitance, degradability, and mechanical properties. After injection in an adult rat SCI model, the ratio of M2/M1 cells in the treatment group was twice that of the control group (Li et al., 2020a), demonstrating that NHC can regulate the phenotype of M2 cells. Moreover, Cornelison et al. obtained injectable hydrogel (IOA) by optimizing the acellular nerves by enzymatic hydrolysis. Immunofluorescence staining was performed on animals treated with phosphate buffered saline (PBS) and injectable optimized acellular (iOA) for 1 week (Figure 1A). Quantitative analysis showed that the ratio of M1/M2 macrophages was significantly reduced by up to three-fold (Figure 1B), demonstrating transformation from pro-inflammatory to anti-inflammatory phenotypes. This change has important significance for the favorable transformation of the immune microenvironment and improvement of spinal cord repair and regeneration (Cornelison et al., 2018).

Oligo [(polyethylene glycol)fumaric acid] (OPF) hydrogels have good biocompatibility and semiconductor properties, and have been used in various types of tissue engineering, including growth factor delivery and cell encapsulation (Zhou et al., 2018b). Hakim et al. showed that OPF is positively charged by copolymerization to form positively charged oligo [(polyethylene glycol) fumaric acid] (OPF+), and the number of local DC206+ cells after implantation in the injury site appears over time, with a growth trend (Hakim et al., 2015).

The polylactide-glycolide nanoparticles (NPs) synthesized by Park et al. have no drug ingredient and no target effect. They can be combined with and reprogrammed by the internalization properties of immune cells after injection into the tail vein of mice. Continuous injection therapy was performed after SCI, and the macrophages at the injured site were quantitatively analyzed on days 7 and 84. No change was observed between the groups (Figure 1C), but the ratio of M2 phenotypic cells to the total number of macrophages was significantly up-regulated in NPs compared with the PBS group (Figures 1D,E). Importantly, the overall immune cells in the injured area decreased by four-fold compared with those in the control group, indicating that NPs may down-regulate pro-inflammatory factors and up-regulate anti-inflammatory factors. Indeed, this conjecture was verified in the quantitative real-time–polymerase chain reaction (qRT-PCR) data. Compared with the PBS group, the key pro-inflammatory markers of the M1 phenotype in the NP group are iNOS and monocyte chemoattractant protein-1 (MCP-1). The expression levels of MCP-1 and CD86 were significantly down-regulated from the 7th day, whereas the levels of the M2 markers IL-10 and CD206 were significantly increased (Park et al., 2019).

#### 2.1.2 Regulation of Cell Polarization by Biomaterials Loaded With Stem Cells

**FIGURE 2:**
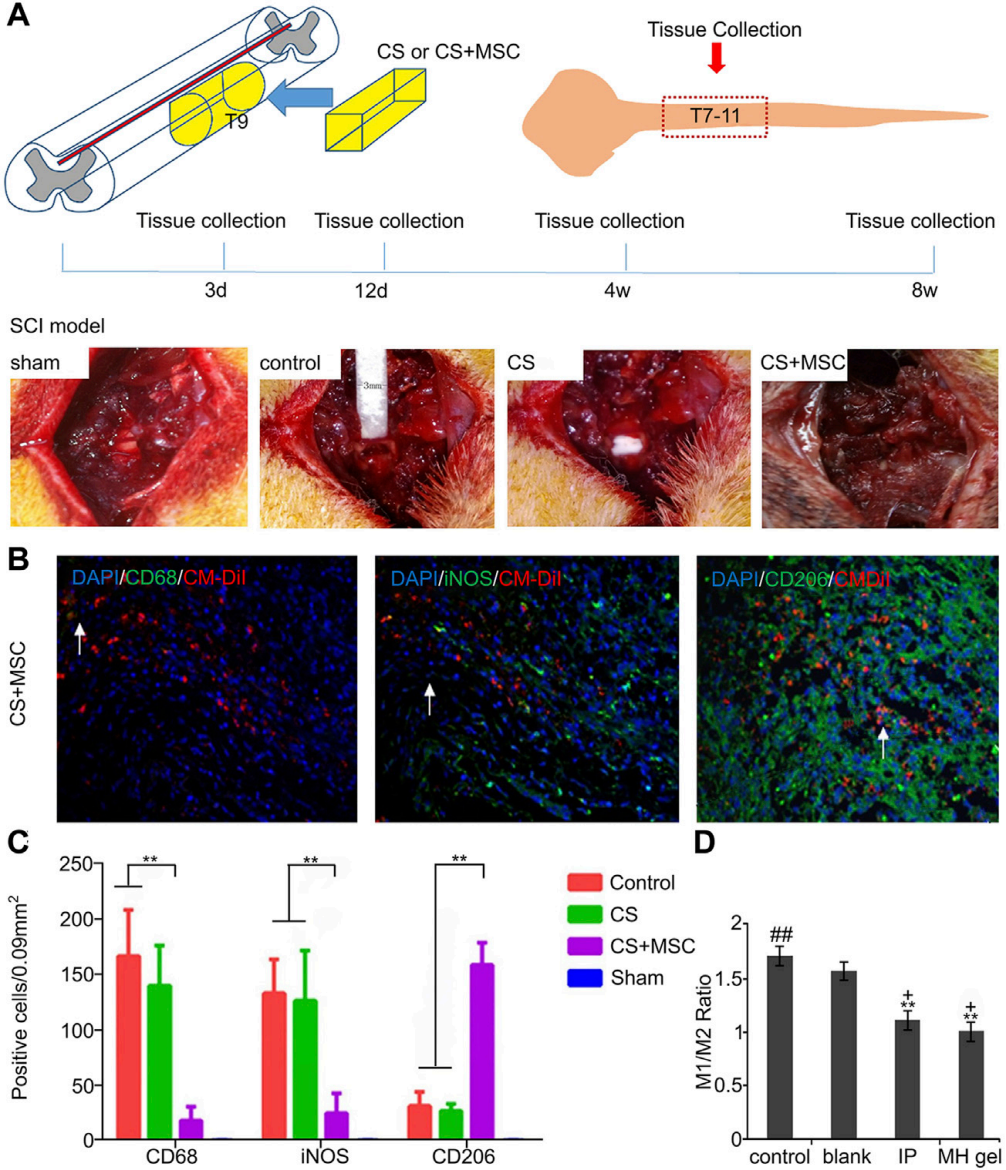
Regulation of macrophages/microglia by biomaterials loaded with different substances. **(A)** SCI model construction, 3 mm segment of left spinal cord of rat T9 was removed, and CS or CS composite MSCs were implanted. **(B)** CD68, iNOS and CD206 immunohistochemical staining of spinal cord slices in the treatment group. **(C)** The bar graph shows the number of CD68, iNOS and CD206 positive cells at 3, 12, and 28 days after surgery. **(D)** M1/M2 ratio of untreated control group, blank gel group, local MH gel group and whole body IP treatment group. Reproduced with permission from (65, 70).

Stem cell transplantation therapy has become a popular treatment for SCI. MSCs can secrete various anti-inflammatory cytokines, such as TNF-β1, IL-13, and ciliary neurotrophic factor (CNTF), neurotrophic factor 3 (NT-3), and IL-10. In terms of neuroprotection, MSCs can release brain-derived neurotrophic growth factor (BDNF), glial cell-derived neurotrophic growth factor (GDNF), nerve growth factor (NGF), and other neurotrophic factors (Csobonyeiova et al., 2019). Therefore, the combination of stem cells and biological materials has become one of the most promising treatment strategies for SCI. A previous study showed that hydrogel scaffolds loaded with bone marrow MSCs act on SCI injury sites by releasing CCL2 to recruit and promote macrophages to polarize toward the anti-inflammatory M2 phenotype. The locomotion ability of the hind limbs in rodents with SCI was significantly improved (Papa et al., 2018). Peng’s experimental team integrated MSCs into a neuro-guided collagen scaffold (CS) and implanted them into a spinal cord hemiectomy model (Figure 2A) and performed immunohistochemical staining analysis (Figure 2B). As a result, more M2 anti-inflammatory cells were observed on days 3, 12, and 28 of treatment in the combined implantation group (Figure 2C), significantly better than that in the control group. This suggests that the complex contributes to the formation of an anti-inflammatory environment and promotes SCI repair (Peng et al., 2018). Mimicking the properties of native ECM is essential for the proliferation and differentiation of encapsulated stem cells, and a cell-adaptive neurogenic (CaNeu) hydrogel scaffold developed by Yuan et al. possesses the dynamic network properties of native ECM. The scaffold loaded with ADSCs can promote M2 macrophage polarization by inhibiting the PI3K/Akt signaling pathway (Yuan et al., 2021). The application of the complex effectively suppressed the inflammatory response, established an anti-inflammatory immune microenvironment, reduced the harmful effects of inflammation, and promoted nerve fiber regeneration.

#### 2.1.3 Regulation of Cell Polarization by Drug-Loaded Biomaterials

Poly-L-lactic acid (PLLA) is a biocompatible, biodegradable synthetic implant that has been used clinically for 30 years (Fitzgerald et al., 2018). A study on an animal model of SCI implanted electrospun PLLA containing ibuprofen and triiodothyronine (T3) into the injury site of rats. Flow cytometry data showed the proportion of M1 macrophages was reduced compared to the untransplanted group (Bighinati et al., 2020). Another study showed that the MoS2@PEG nanoflower composite prepared using the microwave-assisted hydrothermal method has strong drug-carrying capacity and good biocompatibility. Moreover, because of its rich ether and hydroxyl groups, etanercept (ET) can be bound to its surface by noncovalent bonds to facilitate release. *In vitro* and *in vivo* experiments have shown that the combination of ET-MoS2@PEG can regulate the polarization of macrophages to the anti-inflammatory M2 phenotype and inhibit the expression of M1-related pro-inflammatory markers to significantly improve exercise recovery (Sun et al., 2019b). Similar studies have shown that a new type of minocycline loaded in poly-ε-caprolactone NPs (PCL Mino) developed by the phagocytosis of microglia/macrophages can reduce the pro-inflammatory response, and reduce the proportion of M1 polarized cells while preserving that of M2 to improve the SCI microenvironment (Papa, 2017). Minocycline hydrochloride (MH), as a commonly used clinical drug, can be used to treat secondary injuries through its anti-inflammatory, anti-oxidant, and anti-apoptotic properties. However, high concentrations are necessary to achieve neuroprotective effects, but systemic high-dose administration cause liver toxicity and even death (Wang et al., 2019). Therefore, to solve this problem, a new type of dextran sulfate (DS)-MH complex was developed, which is encapsulated in agarose hydrogel to form a drug delivery system. After the system is injected into an adult rat injury model, it can be released at a high dose of 24.73–35.74 μg/ml in the acute phase (1–3 days) to achieve neuroprotection. It can still release the drug at a concentration of 1.00 μg/ml until the 21st day, which is sufficient to regulate chronic inflammation. Moreover, quantification of macrophages/microglia in the injury center showed that the percentage of M1-type cells in the treatment group was significantly reduced (Figure 2D) (Wang et al., 2017a).

#### 2.1.4 Regulation of Cell Recruitment

Collagen is a ubiquitous protein in the human body and represents the most suitable natural polymer scaffold material for SCI repair, with good biocompatibility, and low antigenicity and degradability (Xiao et al., 2018). Snider et al. showed that a type I collagen composite scaffold with micropores can regulate the local inflammatory response by regulating the recruitment of microglia/macrophages at the injury site, laying a good foundation for the recovery of SCI (Snider et al., 2017). A previous study showed that injecting injectable collagen hydrogel containing NT-3 hollow microspheres into a spinal hemisectioned rat model significantly reduced the activity and number of macrophages/microglia in the treatment group compared with the control group (Breen et al., 2016). Biomaterials can reduce excessive immune inflammatory response by effectively reducing the recruitment of immune cells, thus establishing a good microenvironmental state supporting injury recovery and promoting SCI repair.

### 2.2 Regulation of Neutrophils by Biomaterials

**FIGURE 3:**
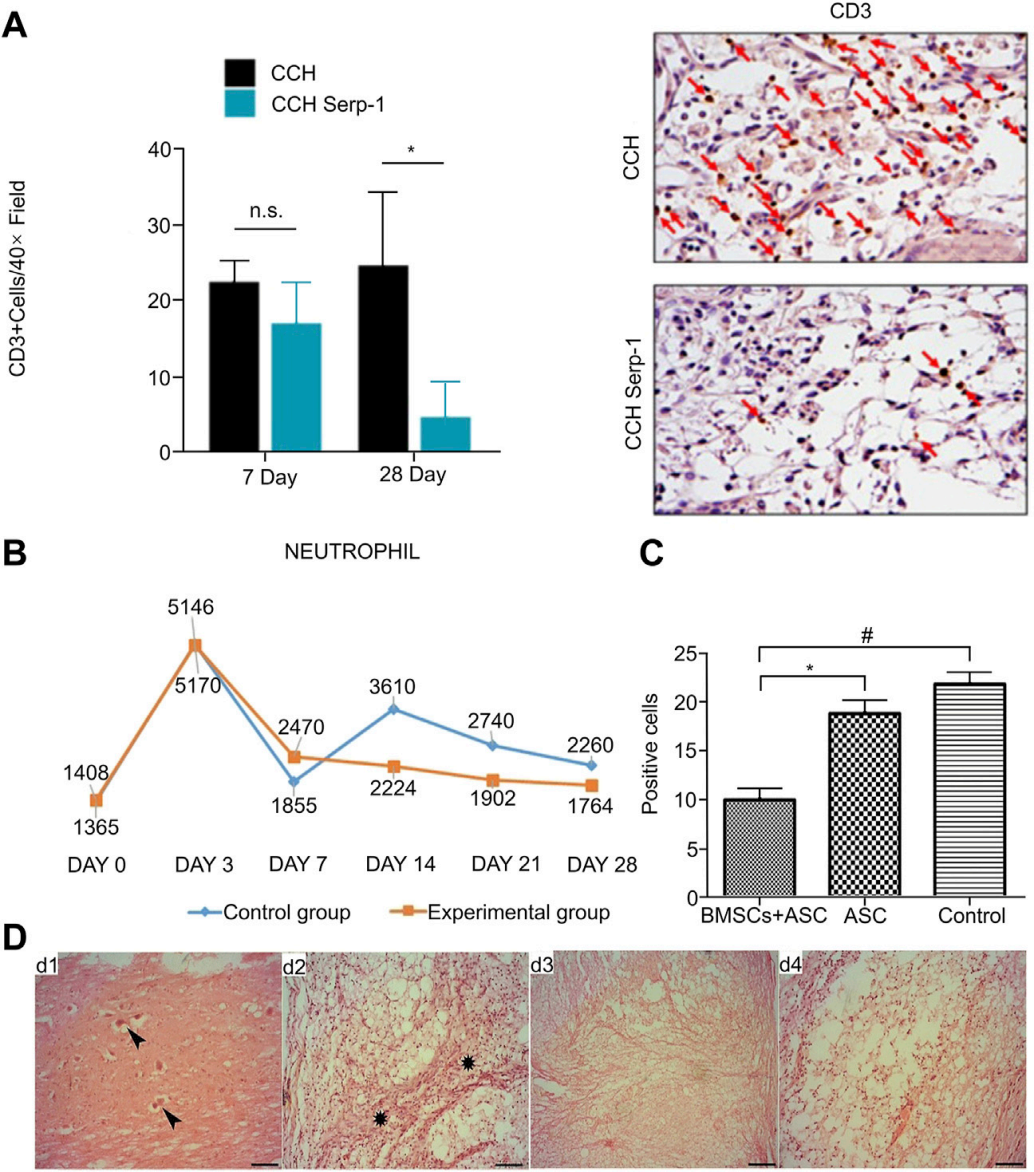
Regulation of neutrophils and lymphocytes by biological materials. **(A)** Quantitative analysis of CD3+ cells in different treatment groups and immunohistochemical staining of CD3+ cells on day 28 under the same conditions. **(B)** Changes of neutrophils (106/L) in the experimental group and the control group at different time periods. **(C)** T lymphocytes (CD5) showing significant differences for the BMSCs + ASC group compared with the ASC group (*p < 0.05) and control group (#*p* < 0.05) at 2 weeks after surgery. **(D)** Histological characteristics of different groups on the 28th day after operation. d1 and d2 were controls: fibrotic tissue infiltration and neuron necrosis were seen. d3 and d4 were experimental groups: the injury site was filled with neatly arranged white matter fibers and mild inflammation. Reproduced with permission from (82, 84, 86).

Neutrophils, as cells of the innate immune system, are the first line of defense against tissue damage and infection, and recruit relevant immune cells by deploying recruitment signals to remove cell debris or pathogens to promote tissue recovery (Kovtun et al., 2018). In SCI, however, excessive recruitment of neutrophils is often considered to cause tissue damage, and destruction of the local microenvironment is not conducive to SCI repair. Neutrophils first arrive at the site of injury in the acute stage of SCI and peak at 24 h. These cells phagocytose and remove cell debris and secrete myeloperoxidase (MPO), metalloproteinase, and elastase, and release ROS (Virginie Neirinckx et al., 2014). These injury-related factors accumulate in the injured area, leading to an aggravated inflammatory response and secondary tissue damage (Zivkovic et al., 2021). Related experimental studies have shown that the myelin sheath in the injured area of MPO-KO mice is more intact, the production of pro-inflammatory factors is significantly reduced, and the exercise recovery ability is significantly better than that of wild-type mice (Kubota et al., 2012). As a proinflammatory factor, leukotriene B4 (LB4) promotes recruitment by binding to LTB4 receptor 1 (BLT1) on neutrophils, ultimately leading to tissue damage (Crooks and Stockley, 1998). Saiwai et al. showed that the degree of neutrophil infiltration in BLT1 knockout mice was significantly lower than that in WT mice, showing better functional recovery (Saiwai et al., 2010). Therefore, biomaterials devoted to the regulation of neutrophil recruitment have a good development prospect. However, there are few experimental articles on the regulation of neutrophils by polymeric biomaterials in the context of SCI. Conversely, tissue damage caused by excessive inflammatory responses to neutrophils treated with biomaterials has been reported in other disease models. In acute ischemic stroke (AIS), Tang et al. found that platelet-mimicking nanoparticles (PTNP) loaded with Piceatannol and superparamagnetic iron oxide were synthesized using the properties of neutrophils interacting with platelets during the inflammatory response. The platelet membrane coating successfully recognizes neutrophils and delivers loaded Piceatannol to cells, thereby reducing the inflammatory response caused by excessive neutrophil infiltration, reducing the infarct area to achieve the therapeutic goal (Tang et al., 2019). Another study found that microenvironmental homeostasis can also be maintained by precise regulation of neutrophil apoptosis. In this experiment, the synthesis of protein nanoparticles NPs coupled with doxorubicin (DOX) (DOX-Hyd-BSA NPs) was shown to selectively target inflammatory neutrophils *in situ*. Subsequently, NPs binding to neutrophils via Fcγ receptors released DOX from neutrophils, in under acidic conditions, thereby inhibiting neutrophil migration and inflammatory responses. Moreover, in a disease model, this complex prevented brain injury during cerebral ischemia-reperfusion and significantly improved survival in sepsis mice without inhibition of systemic immunity (Zhang et al., 2019b). Javdani et al. treated SCI rats with selenium NPs (SeNPs) orally for 28 days, and performed histopathological analysis of the injured sites of experimental and control rats. The results showed more well-arranged white matter fibers and less neuronal necrosis in the experimental group (Figure 3D). Moreover, the inflammatory response in the center of the lesion was significantly reduced and the number of neutrophils was significantly lower than that in the control group (Figure 3B). Therefore, they concluded that nano-selenium can protect the nerves of SCI rats by inhibiting inflammation and can promote the recovery of neuronal function (82). In other areas, biomaterials that regulate neutrophils to reverse the adverse immune microenvironment to promote the treatment of diseases have been well studied, which has led to a promising treatment regimen for SCI.

### 2.3 Regulation of Lymphocytes by Biomaterials

Studies have shown that the infiltration of leukocytes in the peripheral blood into the injured spinal cord is directly involved in destroying the immune microenvironment after SCI and aggravating the cascade of damage, especially T lymphocytes (Esposito et al., 2012). The acellular spinal cord (ASC) scaffold has good biocompatibility, and, after being combined with BMSCs, it was implanted into the spinal cord hemisection model of SCI rats. Immunofluorescence and semi-quantitative analysis showed that the number of DC5-positive T lymphocytes in the scaffold group was significantly lower than that in the control group (Figure 3C). The results demonstrated that the complex can reduce the migration of blood-derived T lymphocytes at the SCI site, reduce the degree of inflammation in the injured site, and promote the recovery of SCI (Wang et al., 2017b). A similar study in chronic experimental autoimmune encephalomyelitis (EAE) involved preventive injection of nanovesicles (NVs) isolated from adipose stem cells (ASC), which was shown to inhibit the activation of T lymph in the inflamed CNS. Cellular transportation has a beneficial effect on chronic EAE (Farinazzo et al., 2018). The Kwiecien research team developed a physical Cs-collagen hydrogel (CCH) with structural stability, excellent biocompatibility, low antigenicity and high biodegradability to continuously deliver immunomodulatory biopharmaceutical Serp-1. After the complex was implanted in the rat SCI model, immunohistochemical staining and quantitative analysis showed that CD3-positive T cells in the injured area were significantly reduced compared with those in the control group (Figure 3A), which greatly improved the local immune microenvironment after SCI and promoted functional improvement (Kwiecien et al., 2020).

### 2.4 Regulation of Astrocytes by Biomaterials

Astrocytes represent the most abundant cell types in the CNS, with main roles including maintaining environmental homeostasis, protecting nerves, and synaptic plasticity (Li et al., 2020b). After SCI, reactive astrocytes gather around the lesion and hypertrophy and proliferation are observed. These reactive astrocytes that migrate centrally to the lesion contribute to tissue repair, however, excessive proliferation eventually leads to the formation of glial scars and inhibits axon growth and regeneration (Okada et al., 2018). Although the production of glial scars inhibits the growth of axons, additional studies have shown that conditional knockout of transcriptional activator 3 (STAT3) in astrocytes leads to down-regulation of GFAP, and glial scars cannot form normally. This leads to the spread of inflammation at the injury site and impaired exercise recovery (Herrmann et al., 2008). Therefore, the favorable balance of the regulation of glial cell activation needs to be further studied to provide a new therapeutic target for functional recovery after SCI.

**FIGURE 4:**
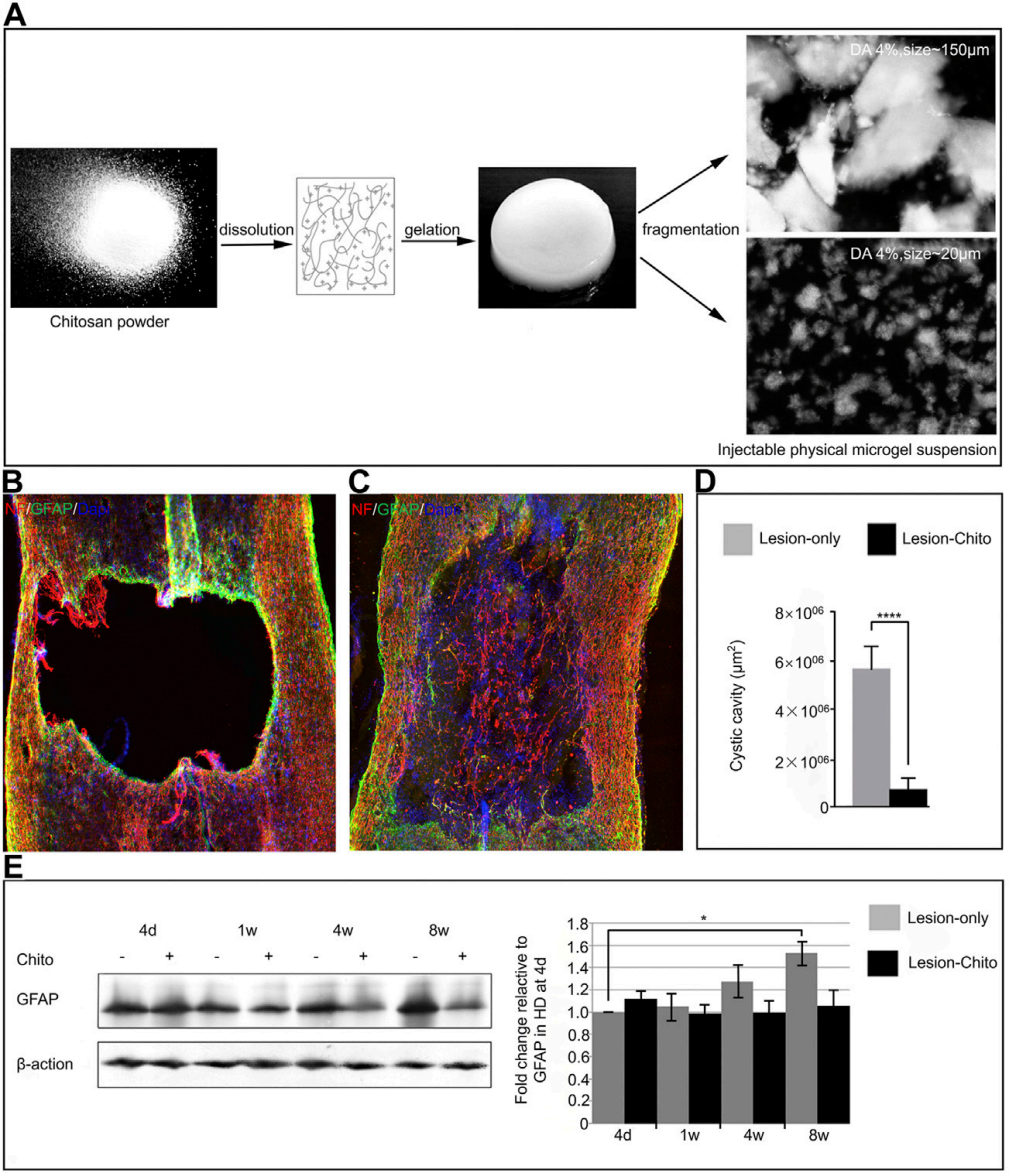
Regulation of astrocytes by biomaterials. **(A)** Preparation of chitosan-FPHS. **(B,C)** Immunofluorescence staining was performed in the control group and the treatment group 4 weeks after surgery. **(D)** Comparison of primary lesion area between the treatment group and the control group. **(E)** Western blot was used to analyze GFAP levels in the treatment group and the control group at different time points after surgery. Reproduced with permission from (56).

After SCI, astrocytes can secrete various chemokines and cytokines to transmit danger signals, worsen the microenvironment of the injured site, and prevent nerve recovery and regeneration (Yang et al., 2020). A recent study showed that the sustained release of dexamethasone sodium phosphate from the PCL/gelatin scaffold can effectively inhibit the proliferation of astrocytes, avoid glial scar formation, and promote axon growth to repair SCI (Rasti Boroojenia et al., 2018). Chelly’s research team designed a scaffold for SCI treatment, which contains Cs and water as a broken physical hydrogel suspension (Cs-FPHS), and has a specific degree of acetylation (DA) (Figure 4A). After implanting the stent into the injury model, immunofluorescence staining was performed on the lesion site at the 4th week, indicating the presence of dense glial scars around the cavity in the control group, while the lesion site in the treatment group was filled with Cs stent (Figures 4B,C). Subsequent quantitative analysis showed that the void area in the treatment group was significantly smaller than that in the control group (Figure 4D). Western blotting analysis of the injured area at different time points after surgery showed that the scaffold significantly reduced the strong proliferation of fibrous astrocytes after SCI (Figure 4E). Surprisingly, western blot quantitative analysis in the treatment group showed that the number of CD206 and Arg-1 positive M2 cells was significantly greater than that in the blank group (Chedly et al., 2017). These findings show that the scaffold can not only inhibit the excessive proliferation of astrocytes, but also regulate the phenotype of macrophages/microglia and improve the immune microenvironment. Similar studies have found that the implantation of CS/MSC scaffolds can significantly reduce the number of GFAP-positive cells and reduce the formation of glial scars (Peng et al., 2018). Compared to synthetic materials, biological scaffolds composed of natural ECM can mimic the complex structure and chemical properties of the extracellular microenvironment *in vivo*. Biological scaffolds possess the following characteristics: natural three-dimensional (3D) structure, ability to promote cell adhesion and proliferation, and biodegradability. The Tukmachev research team injected an ECM hydrogel prepared by decellularization of the porcine spinal cord or porcine bladder into the spinal cord hemisection cavity, and GFAP immunofluorescence staining showed that astrocytes were distributed around the lesion. Moreover, the ECM hydrogel did not migrate to the inside of the lesion, and the Gfap gene expression analysis showed a downward trend in the treatment group (Tukmachev et al., 2016). Thus, the two hydrogels have significant immunomodulatory functions.

## 3 Biomaterial Loading/Inhibition of Cytokines Reverses the Poor Immune Microenvironment

### 3.1 Biomaterials Loaded With Anti-inflammatory Factors

As an effective anti-inflammatory cytokine, IL-10 can inhibit the transcription of TNF and IL-1β mRNA by down-regulating signal transduction by Toll-like receptor related expression of pro-inflammatory genes (Vanderwall et al., 2018; Sabirzhanov et al., 2019). IL-10 is also secreted by macrophages and induces the polarization of macrophages towards an anti-inflammatory phenotype, forming a positive feedback regulation (Lopes et al., 2016). In SCI, IL-10 can provide nutritional support for neurons, reduce secondary inflammation, and improve nerve recovery (Hassanzadeh et al., 2018; Park et al., 2018; Lv et al., 2019). Kastin et al. showed that IL-10 cannot pass through the complete BSCB after being administered peripherally. Only when the BSCB is discontinuous, can IL-10 reach the injured site to achieve the therapeutic effect (Kastin et al., 2003). Therefore, it is critical to develop local implantation or injection therapy based on the combination of biomaterials and cytokines for treating SCI.

Mineral-coated particles (MCM) cultured in specified modified simulated body fluid (mSBF) solutions can greatly improve the efficiency of nonviral transfection of many cell types due to their good bioabsorbability, nanolayer morphology, unique ionic composition, and good dissolution rate (Khalil et al., 2017). Yu et al. demonstrated that the MCM two-factor delivery system loaded with bone morphogenetic protein 2 and vascular endothelial growth factor (VEGF) can effectively promote bone and angiogenesis, and effectively promote the healing of large bone defects (Yu et al., 2014). A study on an animal model of SCI showed that IL-10 loaded MCM was continuously released at the injured site, with *in vitro* test results showing that IL-10 was released for 17 days. This sustained release effect reduced the levels of IL-1β and TNFα and the proportion of M1 macrophages, and increased the number of axons and functional recovery (Hellenbrand et al., 2019). The multichannel PLG bridge has linear channels and interconnected pore structures, such that endogenous cell populations can quickly grow into the interior to fill the damaged area and guide the growth of axons promote functional recovery. Because of its biodegradability, it will eventually be replaced by natural tissue (Pawar et al., 2015; Anzalone et al., 2018). A study showed that 12 weeks after implanting a PLG bridge containing IL-10 and NT-3 bicistronics into a mouse spinal cord hemisection model, immunohistochemistry and quantitative analysis showed that the number of macrophages in the experimental group was three-fold greater than that observed in the control group, and most of them had anti-inflammatory cell phenotype (Smith et al., 2020). The production of damage-associated molecular patterns (DAMPs) activates circulating neutrophils and tissue-resident macrophages to migrate to the site of injury to produce many pro-inflammatory cytokines, aggravating tissue destruction and affecting prognosis. Therefore, it is critical to develop new biomaterials to scavenge DAMPs. Shen et al. developed an immunomodulatory hydrogel scaffold that scavenged DAMPs and slowly released IL-10, which carried a high positive charge and inhibited these immune effects by electrostatically binding and removing DAMPs. Precise gelatin content and porous channel properties result in a well-controlled and sustained release of IL-10, which inhibits microglia activation, reduces pro-inflammatory cytokine secretion, and achieves M2 macrophage polarization (Shen et al., 2022). Its bifunctional immunomodulatory properties regulate the immune microenvironment to facilitate equilibrium. Consequently, secondary injury is avoided, axonal growth and nerve regeneration are accelerated, and motor function recovery is promoted.

**FIGURE 5:**
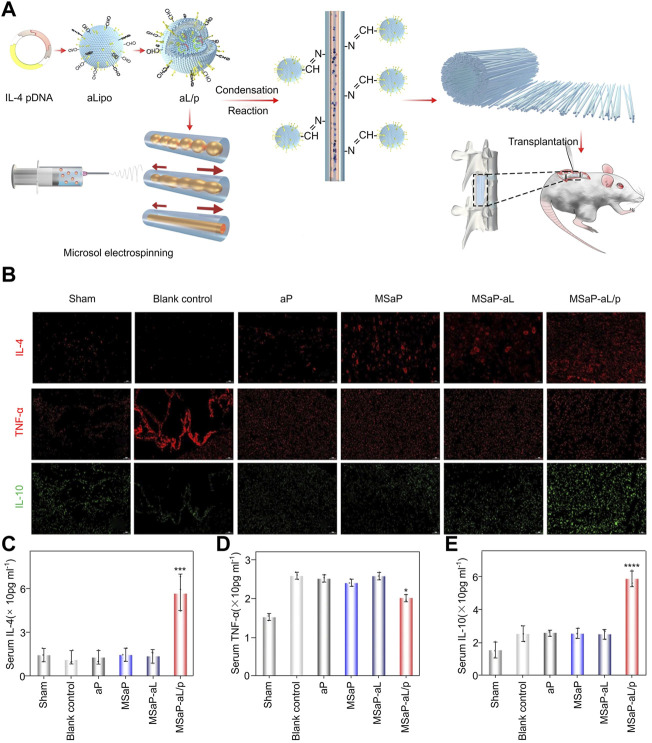
Biomaterials containing anti-inflammatory factors. **(A)** Schematic diagram and application method of composite material. **(B)** Evaluation of immunomodulation of composite materials on the 7th day after operation. ELISA method to detect serum IL-4 **(C)**, tumor necrosis factor-α **(D)** and IL-10 **(E)** levels. Reproduced with permission from (38).

In inflammation in the CNS, interleukin 4 (IL-4) is expressed and plays various regulatory roles (Lee et al., 2010). Inspired, Xi et al. developed an acid-responsive biomimetic fibrous scaffold (MSaP-aL/p) loaded with IL-4 plasmid liposomes, which showed immunomodulatory function and neurogenesis potential based on the polylactic acid production method (Figure 5A). The scaffold can use the acidic environment after SCI to release liposomes and produce a transfection effect and large amounts of IL-4. By immunofluorescence staining, numerous anti-inflammatory factors, including IL-4 and IL10, were shown to be secreted in the injured part of the experimental group and could effectively inhibit the production of TNF-α (Figure 5B). Because of the secretion of many anti-inflammatory factors in the injury site, the serum IL-4 and IL-10 of the MSAP-al/p group were significantly higher than those of other groups, which was confirmed by enzyme-linked immunosorbent assay (ELISA) (Figures 5C,D,E). In addition to secreting anti-inflammatory factors, the complex can induce M2 polarization of macrophages/microglia both *in vitro* and *in vivo*, thereby achieving immunomodulatory effects and inhibiting the inflammatory response in the acute phase. Further research showed that the activated astrocytes and glial scar tissues in the MSaP-aL/p group were significantly less than those in the blank control group (Xi et al., 2020). These findings confirmed that the bionic scaffold has good local immune regulation ability in the acute phase of SCI, can reduce the effect of scar tissue formation in the chronic phase of SCI, and can clear the obstacles for migrating endogenous or exogenous neural progenitor cells to the SCI site.

Similar studies have shown that the prognosis of SCI can be significantly improved by implanting PLG multichannel bridges loaded with lentiviral vectors encoding IL-10 and IL-4 cytokines. The polymer allows axons to pass through, serving as a bridge for axon regeneration and allowing continued release of IL-10 and IL-4 anti-inflammatory factors for up to 12 weeks. Immunofluorescence analysis showed that compared with the blank group, the number of anti-inflammatory M2 macrophages in the test group increased significantly, indicating that the complex can regulate cell polarization. Further studies have shown that IL-10 and IL-4 reduce the release of related inflammatory factors by inhibiting the STAT3 and STAT6 cell pathways, respectively, and improving the immune microenvironment of SCI (Park et al., 2018).

### 3.2 Biological Materials That Inhibit the Release of Pro-inflammatory Factors

The nano-selenium synthesized by Javdani et al. affects immune cells, especially macrophages, and effectively inhibits the release of inflammatory factors, such as COX-2 and TNF-α. The effect of nano-selenium is mainly achieved by blocking the MAP kinase pathway to reduce the endotoxin level of Gram-negative bacteria (Javdani et al., 2019). Among the many drugs used to treat SCI, only methylprednisolone (MP) is clinically effective, however, due to its systemic side effects during the treatment process, the treatment results are unsatisfactory. Karabey-Akyurek et al. loaded the water-soluble MP sodium succinate (MPSS) in biodegradable PCL NPs and dispersed them in the fibrin gel system. After implanting the injured site in a minimally invasive manner, the inflammatory cytokines IL-6 and IL-1β were quantitively analyzed by ELISA, showing significantly lower levels in the treatment group compared with those in the trauma group (Karabey-Akyurek et al., 2017). In an animal experiment that stimulated the intrinsic growth capacity of damaged neurons, Zhang et al. used a 3D fiber-hydrogel scaffold-based drug delivery system combined with MicroRNAs (miRs) and MPs for SCI repair. Subsequent RNA-seq analysis revealed that the expression of pro-inflammatory genes (such as CD74, CCL7, CXCL1, and Ptx3) was significantly down-regulated when MP was present, reducing the inflammatory response and significantly improving functional recovery (Zhang et al., 2021). The appropriate dosage of MP is crucial to the recovery of SCI. To avoid drug toxicity, Yu’s team successfully fabricated an Nap-Phe-Phe-Tyr (H2PO3)-OH (1P) self-assembly system by using the solid-phase peptide synthesis (SPPS) method. After the system is loaded with alkaline phosphatase, it can support the release of MP up to 98% in a sustained manner within 120 h. The complex targeted drug delivery effectively and avoided the toxic effects of vital organs. Moreover, the ELISA analysis showed that pro-inflammatory cytokines (TNF-α, IL-1β) were significantly reduced, while histological analysis showed that SCI was significantly improved (Yu et al., 2020). This establishes a solid foundation for good recovery of SCI. Similar studies were conducted earlier by Kim et al., in which an NP-based drug delivery pathway that was locally and persistently delivered MP to injured spinal cord tissue resulted in significantly reduced pro-inflammatory responses, lesion volume, and improved behavioral outcomes (Kim et al., 2009). The good drug release characteristics and precise targeted drug delivery of the drug delivery system enable an appropriate drug concentration to be delivered to the injury site and provide a good microenvironment for SCI.

### 3.3 Biomaterials Loaded With Growth Factors

**FIGURE 6:**
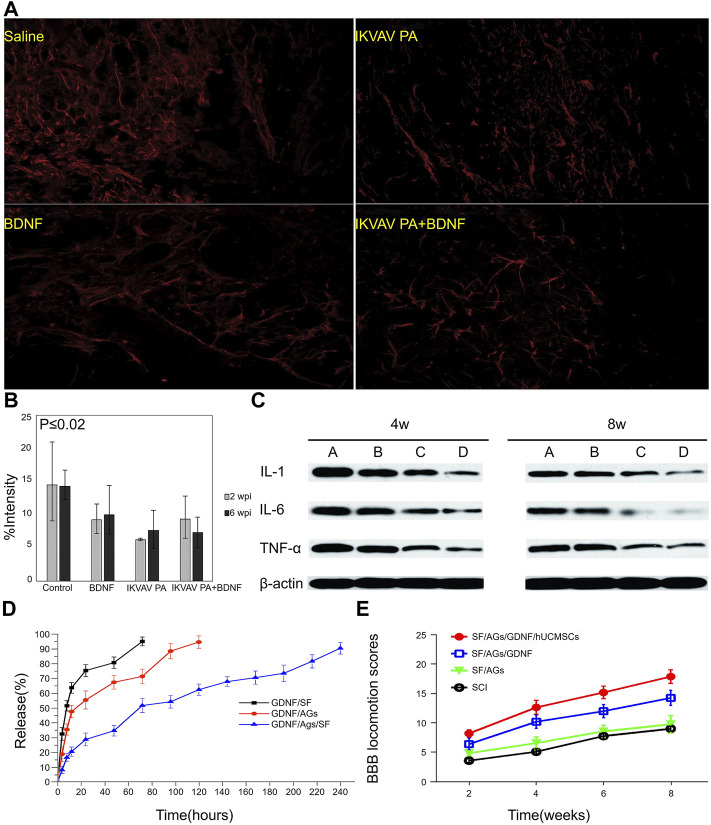
Regulation of immune microenvironment by biomaterials loaded with growth factors. **(A)** Anti-GFAP immunoreactivity in the injured site after 6 weeks of hydrogel treatment in different treatment groups. **(B)** Quantitative analysis of the fluorescence intensity of GFAP staining. **(C)** Western blot detection of inflammation-related markers at 4 and 8 weeks after treatment. A SCI group; B SF/AGs group; C SF/AGs/GDNF group; D SF/AGs/GDNF + hUCMSCs group. **(D)** Scaffold neurotrophic factor release curve. **(E)** Exercise scores of all treatment groups at different time points after treatment. Reproduced with permission from (110, 113).

**FIGURE 7:**
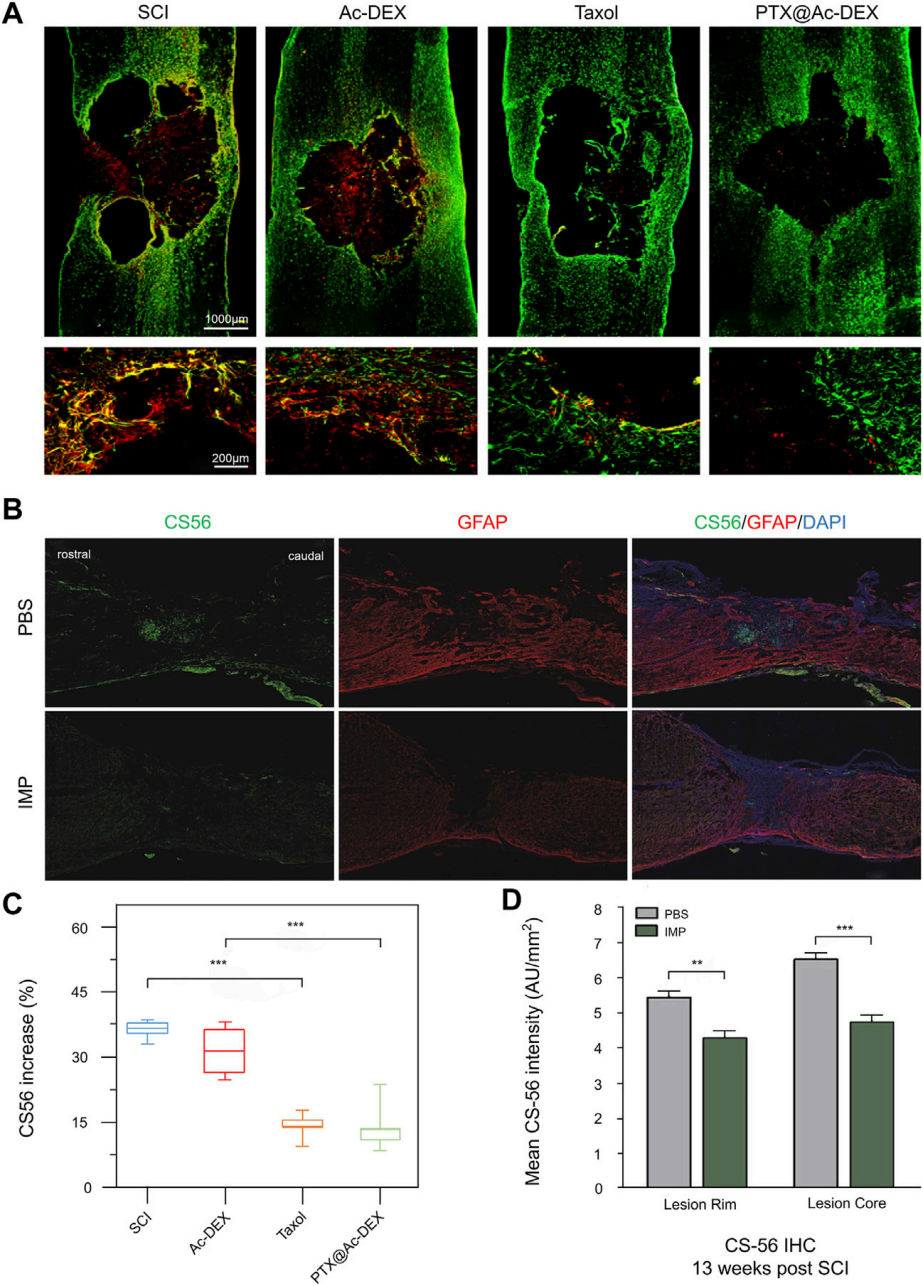
Regulation of undesirable components in extracellular matrix by biomaterials. **(A)** Immunohistochemical staining of CS56 (red) and GFAP (green) at 28 days after injury. **(B)** 13 weeks after SCI, PBS and IMP groups were immunostained with CS-56 and GFAP. **(C,D)** Cs-56 quantitative analysis of lesion area. Reproduced with permission from (119, 124).

Neurotrophic factors protect neurons and promote growth after SCI. However, their clinical transformation results are unsatisfactory due to their poor tissue penetrating ability, seldom can cross the BSCB, resulting in insufficient local release and protein denaturation (Keefe et al., 2017). To solve these problems, neurotrophic factors can be combined with hydrogels through different methods to obtain satisfactory therapeutic effects. Hassannejad et al. synthesized isoleucine-lysine-valine-alanine-valine (IKVAV) functionalized peptide amphiphilic hydrogel (PA) by automated SPPS, and packed BDNF inside. The complex is sustainable while maintaining its activity and is released over a period of up to 21 days. When the fluorescence intensity of GFAP staining was quantified, it was found that the SCI group treated with IKVAV PA hydrogel loaded with BDNF was significantly lower than that treated with normal saline, IKVAV PA hydrogel, and BDNF solution, indicating that the hydrogel loaded with BDNF could reduce the inflammatory response, significantly reduce the proliferation of astrocytes (Figures 6A,B), and strengthen the protection of axons (Hassannejad et al., 2019). As a mature biopolymer, silk fibroin (SF) is widely used in tissue engineering because of its good immunogenicity, excellent mechanical properties, and nontoxicity (Li et al., 2019b). GDNF is necessary for nervous system development and has been proven to promote axon regeneration and functional recovery (Zhong et al., 2020). Because of its sustained release at the local injury site and other issues, it remains a significant challenge to realize the clinical application of GDNF. Therefore, Jiao et al. used SF as a carrier to manufacture a bionic stent (SF/AGS/GDNF) equipped with GDNF, which showed a good sustained-release effect (Figure 6D). Moreover, immunoblotting demonstrated that the inflammatory response markers IL-1β, IL-6, and TNF-α were significantly lower than those in the blank group (Figure 6C). Furthermore, the scaffold effectively improved the immune microenvironment, significantly improved motor function (Figure 7E), and promoted SCI recovery (Jiao et al., 2017). NT-3 in the CNS is deemed a classic nutritional factor, which plays a key role in axon growth and cell survival and differentiation (Keefe et al., 2017). Li et al. implanted the NT-3/SF particle-encapsulated gelatin sponge (NF-GS) scaffold into the rat SCI model, and the results of immunohistochemistry showed that the number of IBA-1-positive macrophages/microglia was significantly reduced, which, in turn, significantly inhibited inflammation (Li et al., 2018). Therefore, the combination of biomaterials and cytokines has a good development prospect for treating SCI.

## 4 Biological Materials Remove Undesirable Components From the ECM and Reverse Poor Immune Microenvironment

The ECM is responsible for regulating and maintaining the mature development and normal function of nerve tissue. After SCI, the ECM reorganizes and participates in key secondary events, including inflammation, gliosis, cell survival, axon growth, neural plasticity, and vascular remodeling (Wiese and Faissner, 2015). The destruction of ECM is the main occurrence of secondary nerve tissue injury. At the site of injury, resident and peripheral inflammatory cells secrete MMPs and ECM components, which are rich in chondroitin sulfate proteoglycans (CSPGs). This causes disruption of the immune microenvironment and inhibits axon growth by receptor protein-tyrosine phosphatase, NOGO receptors NgR1 and NgR3, epidermal growth factor, and leukocyte common antigen associated phosphatase (Haggerty et al., 2017). Abnormal expression of MMPs is involved in various CNS diseases, including stroke and traumatic brain injury. Moreover, the involvement of MMPs in the destruction of the blood-brain barrier or BSCB can promote immune cell infiltration, increase tissue damage, induce cell apoptosis, and ultimately hinder functional recovery after injury (Mirzaie et al., 2018).

### 4.1 Biomaterials Reverse the Poor Immune Microenvironment by Reducing CSPGs

CSPGs are the main factors responsible for inhibiting axon growth and the limited functional recovery after SCI. Studies have shown that down-regulation of CSPGs contributes to the regeneration of axons and the restart of endogenous repair after SCI, such as myelination (Dyck et al., 2018). To promote the growth and regeneration of axons after SCI, it is particularly important to reduce the fibrotic scars caused by the aggregation of CSPG. Jeong et al. intravenously injected biodegradable poly (lactide-ethyllactide) copolymer (PLGA) immunomodified NPs (IMPS) into mice. Thirteen weeks after injury, immunohistochemical staining of the lesions showed that the treatment group had fewer CSPG deposits than the control group (Figure 7B), which was further verified by quantitative analysis (Figure 7D). Surprisingly, the CSPG at the injured site remained at a low level 10 months after SCI, and the reduction of CSPG deposition was more conducive to axon growth and regeneration (Jeong et al., 2017). In a similar study, a novel HA scaffold with a long axis multitube conformation was mixed with PLGA microspheres containing BDNF and VEGF and implanted into an SCI rat model. Semi-quantitative immunofluorescence showed that CSPG deposition in the scaffold group was reduced compared to that in the control group (Wen et al., 2016). Systemic therapy of estrogen after SCI is effective in inhibiting glial scarring and the inflammatory cascade, although side effects prevent its approval. Therefore, the Cox team developed a fast-release nanogel patch (PLGA-E2) based on an agarose gel and it loaded with 17ß-estradiol (E2) for topical application. After use, the E2 concentration at the injury site was twice as high as that of plasma because of its local release characteristics. Luxol fast blue staining showed that the level of CSPG in the lesion area was significantly lower than that of the control group, which is more conducive to the regeneration of the injured and adjacent axons (Cox et al., 2020). Another study found that the transplantation of linear ordered collagen scaffolds (LOCS) and human placental-derived MSCs into completely transected beagle dogs promoted hind limb motor function recovery. Histological analysis showed that the expression of CSPGs was significantly decreased in the treatment group (Han et al., 2018). Similar studies have found that implanting an LOCS-based cetuximab delivery system into the canine SCI site significantly reduced the deposition of CSPG in the lesion site (Li et al., 2017). Moreover, Liu’s research team prepared PTX NPs (PTX@Ac-DEX) by incorporating paclitaxel (PTX) into acetylated dextran (Ac-DEX) NPs. Immunostaining and semi-quantitative results of CSPG showed that PTX@Ac-DEX inhibited the deposition of CSPG in the diseased area (Figures 7A,C), promoting the growth of nerve axons and recovery of movement (Liu et al., 2018).

### 4.2 Biomaterials Inhibit MMPs and Reverse the Poor Immune Environment

As proteolytic enzymes, MMPs can degrade almost all protein components in the ECM, including the destruction of the BSCB in the early stage of SCI. This initiates myelin degradation and macrophage infiltration, and participates in secondary inflammatory events, such as aggravating the permeability of the BSCB, neutrophil infiltration, and macrophage phagocytosis. Additionally, MMPs are important links in inhibiting the formation of glial scars (Miranpuri et al., 2019). MMPs can be used as effective therapeutic targets to improve the immune microenvironment after SCI and to promote nerve growth, axon regeneration and functional recovery. Zhang et al. prepared an AST-PCL (polycaprolactone) membrane that stably releases astragaloside IV (AST). The SCI rat model showed reduced secretion of MMP-9 and inhibition of neutrophil infiltration, reduced inflammatory response, and improved functional recovery in the treated group (Zhang et al., 2019c). MMP responsive copolymer treatment of SCI also has been reported, however, this experiment did not target MMP for therapeutic purposes, but rather used the enzymatic hydrolysis properties of MMPs to prepare a reactive bivalirudin-HPMA copolymer and loaded the polymer into hyaluronic acid and methyl cellulose hydrogel for local delivery. The results showed that the hydrogel system reduced the long-term scar formation and immediate inflammatory response, and improved functional recovery after nerve trauma (Chu et al., 2015). An interesting experiment showed that imidazole-poly (organophosphazene) (I-5), a rapidly degradable hydrogel with thermosensitive sol-gel transition behavior, could be combined with Histamine receptor binding on macrophages interacts to up-regulate MMP-9 expression in cells, promote fibronectin (FN)-rich ECM remodeling, eliminate cysts formed after SCI, and promote tissue repair (Hong et al., 2017).

## 5 Biomaterials Reverse the Adverse Immune Microenvironment by Reducing the Production of ROS

Mitochondria, as important organelles in eukaryotic cells, produce the energy necessary for cell survival, and, as a result, are closely related to the regulation of apoptosis (Jia et al., 2018). However, after SCI, the spinal cord is in a state of ischemia due to local vascular damage and lack of glucose and oxygen supply, so the mitochondria produce a series of biochemical changes. For example, when the electron transport chain (ETC) and mitochondrial membrane potential are altered, the ability of mitochondria to produce adenosine triphosphate (ATP), the cell’s main energy source, is blocked (Scholpa and Schnellmann, 2017). As a result, ATP-dependent cells lose their function, leading to oxidative stress and calcium overload (Hill et al., 2017). Excessive ROS production plays an important role in the cascade of SCI secondary injury (Lin et al., 2021). Excessive ROS production can lead to severe lipid peroxidation, followed by oxidative damage to DNA and proteins, demyelination and degeneration of nerve fibers at the damaged site, and even neuronal apoptosis (Coyoy-Salgado et al., 2019; Kang et al., 2020). Therefore, it is important for functional recovery to reverse the adverse immune microenvironment after SCI by reducing the production of ROS.

Numerous studies have developed and applied various polymer-based biomaterials to inhibit SCI secondary damage caused by oxidative stress. For example, hyaluronic acid (HA), PEG, and Cs, all of which have antioxidant function, can be used as basic components in scaffolders. As an important component of the ECM, HA plays an indispensable role in the support of local structure and the regulation of cell function (Ding et al., 2019). Because of its unique biocompatibility, hydration, lubricity, and specific viscoelasticity, HA has a certain application value in medicine, food, cosmetics and other fields (Avadhani et al., 2017). Under physiological conditions, HA clearance is primarily mediated by enzymes; however, when the body suffers from certain diseases, excessive free radicals can also lead to the degradation of HA. On the contrary, HA can also scavenge free radicals. HA can be used alone or in combination with other polymers to prepare implanted materials, which play an important role in the antioxidant therapy of SCI. PEG, a hydrophilic polymer, can shut down damaged cells by rapid membrane resealing, reducing mitochondrial production of excessive ROS, and reducing further damage to spinal cord tissue (Luo et al., 2002). As noted, Cs is a type of natural polysaccharide polymer, with properties including biocompatibility, biodegradability, reproducibility and nontoxicity. Cs also has unique anti-inflammatory, antibacterial, and anti-microbial properties (Yao et al., 2018). Additionally, Ivanova et al. showed that Cs and its derivatives with REDOX regulatory activity can inhibit free radical-mediated cellular oxidation through their scavenger effect on cellular free radicals, thereby enhancing intracellular antioxidant enzyme activity and preventing ROS production and lipid peroxidation (Ivanova and Yaneva, 2020). However, the antioxidant effects of single polymer biomaterials are relatively limited. To compensate for this, various therapies combining drugs with biomolecular materials have emerged, including minocycline, glutathione, vitamins, and melatonin wrapped in scaffolds to protect against oxidative stress (Courtine and Sofroniew, 2019; Morgado et al., 2019; Zimmermann et al., 2021). Based on the polysialic acid (PSA) sustained-release delivery system, minocycline can be loaded and administered continuously for up to 75 h. When injected into the injured site, anti-inflammatory and antioxidant effects were significantly improved, and the survival rates of myelin and axon were improved (Wang et al., 2019). Qian et al. found that a melatonin/PCL neural catheter-controlled release scaffold corrected mitochondrial function and reduced oxidative stress, thus improving the immune microenvironment and promoting nerve fragment clearance and neuron proliferation (Qian et al., 2018). Notably, the combination of some metal inorganic materials with antioxidant function and polymer biomaterials has been widely used in antioxidant therapy of SCI models.

### 5.1 MnO2-Loaded Biomaterials Reduce the Production of ROS

Manganese (Mn) is an essential nutrient element in the human body and participates in various cellular functions. Indeed, manganese as a cofactor can maintain the activity of manganese superoxide dismutase (Mn-SOD) (Pan Chen and Aschner, 2018). The antioxidant effect of Mn plays a vital role in maintaining the homeostasis of the immune microenvironment and the regulation of neuronal activity after SCI. However, excessive Mn accumulation cause Mn poisoning, which is caused by Mn ions interfering with calcium homeostasis, leading to mitochondrial dysfunction and increasing the production of ROS (Luo et al., 2021). Therefore, it is particularly important to find an appropriate therapeutic dose to avoid poisoning caused by excessive Mn accumulation.

**FIGURE 8:**
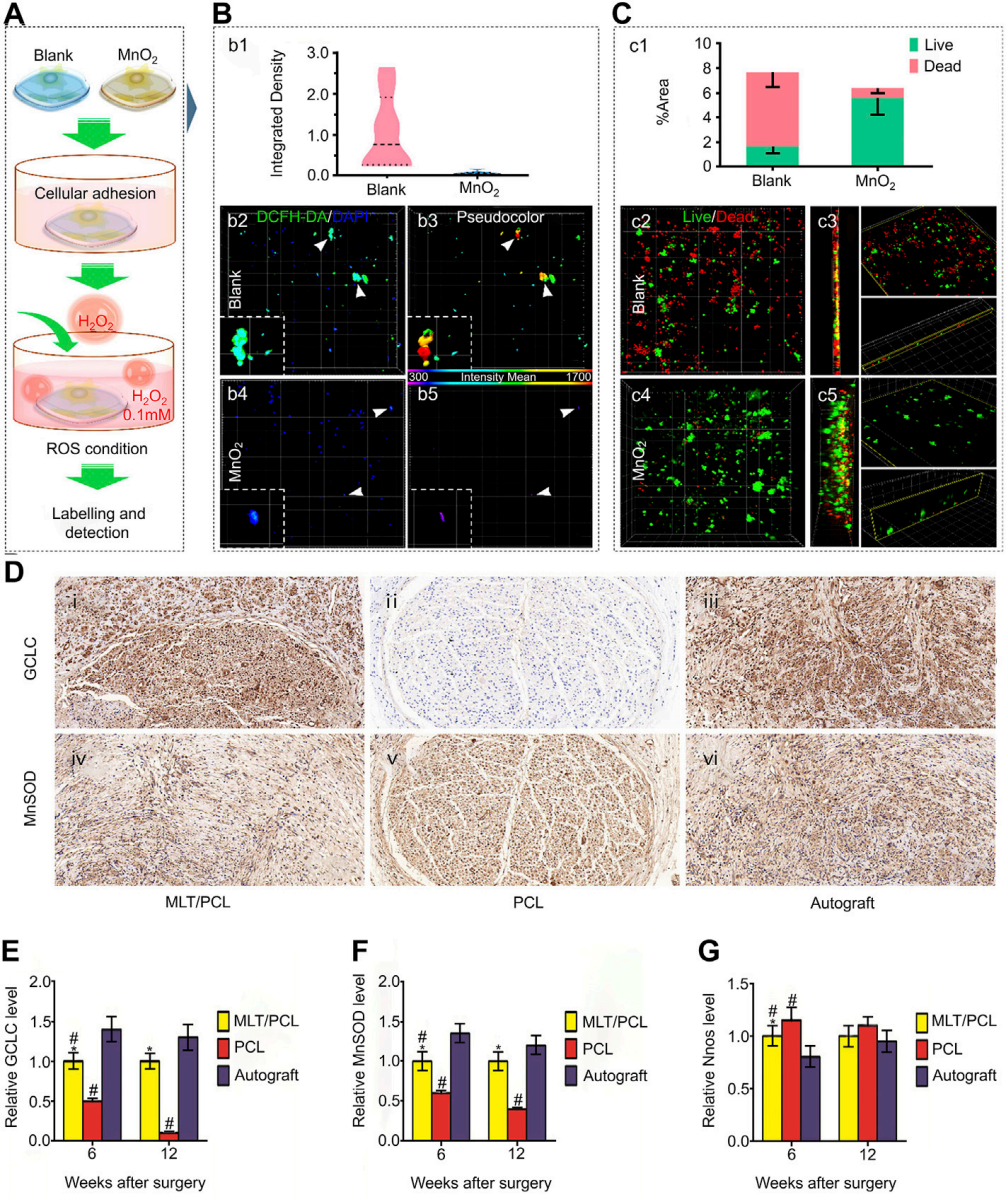
Biomaterials reverse the adverse immune microenvironment by removing harmful components from the extracellular matrix. **(A)** Simulate the cultivation of MSCs in a microenvironment containing ROS. **(B)** After labeling with dichlorodihydrofluorescein diacetate (DCFH-DA), quantify (b1) intracellular ROS levels in blank (b2, b3) and MnO2 NP points (b4, b5) hydrogels. **(C)** Use live/dead assay to analyze cell survival in the blank group (c2, c3) and treatment group (c4, c5), and quantify the stained area (c1). **(D)** Oxidative stress assessment of MLT/PCL and PCL catheters after surgery. **(E)** Relative level of GCLC. **(F)** Relative level of MnSOD. **(G)** Relative level of nNOS. Reproduced with permission from (139, 142).

Li et al. prepared a spot-like hydrogel with antioxidant effects and dispersed nano-manganese dioxide (MnO2 NP) in polypeptide-modified hydrogel (Figure 8A). The complex has a suitable therapeutic dose of Mn ions and excellent low cytotoxicity characteristics. In a test to detect the neutralization effect of ROS, compared with blank hydrogel, the hydrogel containing MnO2 NP significantly reduced the H2O2 content after incubation for 1 and 2 h, and the subsequent cell test and quantitative analysis showed significantly reduced intracellular ROS (Figure 8B). Biomaterials should have good biocompatibility and low cytotoxicity to promote the bridging growth of MSCs and neural tissue. This was confirmed in the subsequent dead/alive staining test, in which the dead cells were widely distributed in the blank hydrogel group, whereas only a small proportion of dead cells were observed in the MnO2 group (Figure 8C) (Li et al., 2019c). The emergence of this combination has made an important contribution to maintaining the homeostasis of the immune microenvironment after SCI.

### 5.2 Selenium-Containing Biomaterials Reduce ROS Production

Selenium, as an essential trace element in the human body, is an important antioxidant with a strong antioxidant function. Bioactive selenoproteins can inhibit lipid peroxidation, block or reduce the chain reaction of lipid peroxidation, and reduce the excessive production of ROS (Chen et al., 2015). Rao et al. designed and synthesized a novel tetramethylpyrazine (TMP)/monosialotetrahexosylganglioside (GM1)-loaded polysaccharide protein complex/PG-6 polypeptide-modified multifunctional SeNPs, which inhibits ROS overproduction by inhibiting the activation of p53 and MAPK pathways, thereby preventing mitochondrial dysfunction (Rao et al., 2019). Another study showed that the synthesis of water-soluble selenium quantum dots (Se-CQDs) by hydrothermal treatment of L-selenocysteine can scavenge ROS. The oxidative damage of astrocytes, N2a, and PC12 cells by H_2_O_2_ was significantly reduced following *in situ* injection, and the cell activity was greatly improved. Simultaneously, the fluorescence intensity of DCF was significantly lower in the treatment group than that in the control group, further confirming that Se-CQDS can effectively reduce the ROS levels (Luo et al., 2020).

### 5.3 Zinc-Loaded Biomaterials Reduce ROS Production

Zinc is a powerful antioxidant that reduces oxidative stress and has neuroprotective effects (Heller et al., 2020). A lack of zinc in mitochondria can lead to apoptosis of nerve cells. Studies have shown that zinc, as an important structural component of NOS, can inhibit the catalytic activity of NOS, down-regulate the expression of inducible nitric oxide synthase (iNOS), and reduce the activation of the iNOS promoter mediated by cytokines, thereby reducing oxidative damage (Li et al., 2020c). The combination of zinc and biomaterials to reduce oxidative stress in SCI and promote neurological recovery has been rarely studied, but has been investigated in the context of other diseases.

In a study on the treatment of limb ischemia, the designed zinc-based metal-EGCG capsule (EGCG/Zn-PS) was shown to promote angiogenesis, cell proliferation and limb blood perfusion recovery by removing excessive ROS in the ischemic microenvironment through the slow release of Zn^2+^ (Chen et al., 2021). Ahmed et al. found that nanofiber pads prepared by Cs, polyvinyl alcohol and zinc oxide have strong antioxidant activity, antibacterial potential, biocompatibility and biodegradation, and could effectively promote wound healing in patients with diabetes (Ahmed et al., 2018b).

### 5.4 Antioxidant-Loaded Biomaterials Reduce ROS Production

In the second stage of SCI, excessive ROS production leads to degeneration and apoptosis of nerve cells, affecting functional recovery. Therefore, neutralizing excessive ROS through antioxidant enzymes has become an effective treatment strategy. Copper/zinc superoxide dismutase (SOD1) reduces ROS cascade damage by converting radical superoxides into molecular oxygen and H_2_O_2_ through REDOX reactions (Eleutherio et al., 2021). Nukolova et al. first combined SOD1 with a polycation to form a polyionic complex, which was then coated with the anionic block copolymer PEG-polyglutamate (bilayer [DC] nanase) to improve motor function recovery in SCI rats by neutralizing excessive ROS (Nukolova et al., 2018). As an antioxidant, melatonin protects nerves from damage by scavenging free radicals and intermediates of oxygen and nitrogen, and enhances antioxidant enzyme activity. Reducing the free radical production by preventing electron leakage in the mitochondrial ETC is another of its antioxidant mechanisms (Dilek Taskiran, 2000). Qian et al. took advantage of this feature to produce a melatonin/polycaprolactone neural catheter scaffold by 3D printing; this scaffold released melatonin in a controlled manner to reduce oxidative stress and mitochondrial dysfunction and improve the immune environment. *In vitro* testing demonstrated that the ROS level in the MLT/PCL group was significantly lower than that in the PCL group by flow cytometry. In subsequent experiments, the therapeutic effect was evaluated *in vivo*. Several markers related to oxidative stress in the damage center were tested. The results showed that the expression of NOS in the treatment group was significantly lower than that in the control group, whereas glutamine acid cysteine ligase and MnSOD were both higher than those the control group (Figures 8D–G) (Qian et al., 2018). The production of these innovative scaffolds holds great significance to neural engineering, especially in the context of SCI. These scaffolds not only can increase nerve expression, cell proliferation, and promote *in vivo* function, electrophysiology, and morphological nerve regeneration, but also can improve the immune environment by reducing oxidative stress, inflammation, and mitochondrial dysfunction to promote the recovery of SCI function.

## 6 Conclusion and Prospects

SCI is a serious injury of the CNS, which has attracted increasing attention because of its increasing incidence. Although the medical community has developed various treatment methods for SCI, including surgical anastomosis, surgical decompression, drug therapy, local freezing, physical rehabilitation, and enzyme preparation, there remains no effective treatment. At present, the key problems to be solved in the field of SCI repair include how to effectively regulate the immune microenvironment in the early stage of SCI, inhibit secondary injury, rescue damaged cells and reconstruct the spinal cord microstructure in the late stage of SCI, and promote the growth of axons and regeneration of nerve cells.

This paper reviews the application of polymer biomaterials for treating SCI. Polymers have good biocompatibility, biodegradability, immunogenicity, and linear structures. Biological materials can provide support for the axon regeneration platform, realize local or slow drug release, ensure sustained release of cytokines, regulate cell polarity, increase or decrease inflammatory activity, reduce the local production of ROS, remove undesirable components of the ECM, promote angiogenesis, and inhibit the formation of glial scar. These polymers have been used as scaffold materials in neural tissue engineering. However, no stent can be widely used for treating SCI in the clinic and research on natural polymer materials as scaffold materials has limited development. Therefore, the modification of existing materials and exploration of more advanced materials will advance its therapeutic use. Recently, the research on immune microenvironment-responsive biomaterials after SCI has become a hot research topic given their ability to regulate the adverse immune environment directly or indirectly. For example, polymeric biomaterials can regulate the polarization and recruitment of immune cells alone or in combination with stem cells, biological factors, drugs, and inhibitors and can remove harmful components such as inflammatory cytokines ROS from extracellular matrix. These methods have shown good therapeutic effects in neural tissue engineering.

Much work remains to be done to achieve better repair and recovery of injured spinal cords. For example, the regulation mechanism of biomaterial macrophages and astrocyte polarization needs to be further studied. Moreover, relevant articles show that pro-inflammatory immune cells also play an equally important role in the injury repair stage, so the balance between beneficial and harmful plastic cells is particularly important. Although neutrophils can be an effective therapeutic target for SCI, no relevant biomaterials have been developed for this purpose. Lmw-ha and Tenascin-C, as damage-related molecular patterns (DAMPs), increase the destructive inflammatory cycle at the site of injury, and thus clearance of DAMPs may represent an effective therapeutic target, although related biomaterials have not yet been identified. Currently, some studies have made some progress, but most are still in the preclinical stage (animal or *in vitro*) and treatment for SCI is still a long way off. We believe that the application of polymer biomaterials for treating SCI will eventually achieve good clinical results.
